# Maternal healthcare use by women with disabilities in Rajasthan, India: a secondary analysis of the Annual Health Survey

**DOI:** 10.1186/s40748-023-00165-1

**Published:** 2023-09-04

**Authors:** M. Tara Casebolt, Kavita Singh, Ilene S. Speizer, Carolyn T. Halpern

**Affiliations:** 1 Department of Maternal and Child Health, Gillings School of Global Public Health, University of North Carolina, Chapel Hill, NC USA; 2 Global Public Health and the Common Good Program, Boston College, 140 Commonwealth Ave, Chapel Hill, MA 02467 USA; 3 Carolina Population Center, University of North Carolina, Chapel Hill, NC USA

**Keywords:** Disability, Maternal health, Healthcare use, Accessibility

## Abstract

**Background:**

Women with disabilities face a number of barriers when accessing reproductive health services, including maternal healthcare. These include physical inaccessibility, high costs, transportation that is not accessible, negative attitudes from family and healthcare providers, and a societal belief people with disabilities shouldn’t be parents. While qualitative studies have uncovered these barriers, there is limited quantitative research to determine their effect on use of maternal health services. This study aims to analyze associations between disability and maternal healthcare use among married women in Rajasthan.

**Methods:**

This study is a secondary analysis of the Indian Annual Heath Survey first wave data from 2011. The sample includes 141,983 women aged 15–49 who had given birth between 2007 and 2009. Logistic regression was used to assess the association between disability and use of antenatal, delivery, and postnatal care. Stratified models were created to analyze difference based on birth order of the pregnancy and whether the woman’s place of residence is rural or urban.

**Results:**

The prevalence of disability was 1.23%. Attending at least three antenatal care visits was reported by 50.66% of the sample, skilled delivery use by 83.81%, and receiving postnatal care within 48 h of birth by 76.02%. In the regression model, women with disabilities were less likely to report attending the minimum antenatal care visits (OR = 0.84; CI: 0.76, 0.92). No association was found between disability and skilled delivery or postnatal care. Once the sample was stratified by birth order, women with disabilities reporting their first birth were more likely to report receiving postnatal care than women without disabilities (OR = 1.47; CI: 1.13, 1.91).

**Conclusion:**

Additional research is needed to determine use of maternal healthcare among women with disabilities in India. Maternal services need to be assessed to determine their accessibility, especially regarding recent laws requiring accessibility.

**Supplementary Information:**

The online version contains supplementary material available at 10.1186/s40748-023-00165-1.

## Background

Around the world, an estimated 1 in 5 people have a disability [1]. Despite this, there is a lack of focus on people with disabilities (PWD) in maternal health research. However, there is evidence women with disabilities (WWD) are discriminated against when accessing maternal healthcare as a result of a lack of accessibility, high costs, and a lack of training regarding disability among providers [2–4].

Based on data from the 2011 Census, there are approximately 26.8 million PWD in India, or 2.2% of the population [5]. However, disability advocates and researchers assert this is an underestimation of actual prevalence of disability [6–8]. In part, this is because in India disability is legally defined based on specific diagnoses that fall within general categories of the functions most affected. This study employs the Annual Health Survey in which disability was categorized as mental, visual, hearing, speech, locomotor, multiple, and other disabilities [9].

There are a number of factors that affect use of maternal healthcare in India. Rural women and women from low-income families are less likely to receive antenatal care (ANC), have a facility delivery, and obtain postnatal care (PNC) than high-income and urban women [10]. Women in their late 20 s and 30 s are more likely to deliver at a health facility than younger women. Women who have more children are less likely to deliver at a health facility. Hindu women have higher odds of institutional delivery than women from other religious groups [11]. One of the most significant determinants is education, with the odds of using maternal healthcare increasing as education increases [12].

Maternal healthcare use for India is reported in the National Family Health Survey (NFHS), 2015–2016. In Rajasthan, the site of this study, 85.5% of women age 15–49 years who gave birth in the five years before the survey reported attending at least one ANC visit. Only 38.5% of these women reported attending the WHO recommended four ANC visits [13]. Delivery in a health facility was reported by 84% of Rajasthani women, and 86.5% reported a delivery by a skilled provider. PNC within 48 h of the birth was reported by 64.9% of Rajasthani women in the NFHS [14].

Qualitative studies have found Indian WWD experience many barriers when accessing maternal healthcare. These include limited access to transportation, inaccessible infrastructure, negative attitudes from providers, and a lack of support from community and family members [15]. These barriers all complicate the process of reaching and receiving maternity services for WWD in India.

The aim of this study is to estimate the association between disability status and maternal healthcare use among married women of reproductive age in Rajasthan, India. This includes analysis of attendance of the Government of India minimum recommended number of three or more ANC visits [16], use of skilled delivery, and receiving PNC within 48 h of delivery. Rajasthan was selected because it had a larger prevalence of disability than other states included in the dataset, increasing estimate precision.

## Methods

This study has received ethics review and was determined to be exempt based on the use on de-identified secondary data.

### Dataset

The dataset used for this study is the Indian Annual Health Survey (AHS). This is a longitudinal survey that was conducted by the Ministry of Health and Family Welfare and Census Commission of India over three waves from 2010–2013. Disability data were included in this survey, though the primary purpose was to collect data regarding infant, child, and maternal health. This study only uses data from the 2010–11 baseline survey because we were unable to identify a variable to link participants across the three waves of data collection. In addition, the AHS did not explain how it managed loss to follow-up in later waves [9]. There were four survey tools used to collect data for the AHS. Ever married women ages 15–49 who had given birth between 2007 and 2009 completed a survey regarding their maternal healthcare, pregnancy outcomes, and infant care. Data from this survey used in this study [9].

### Sample

**Fig. 1:**
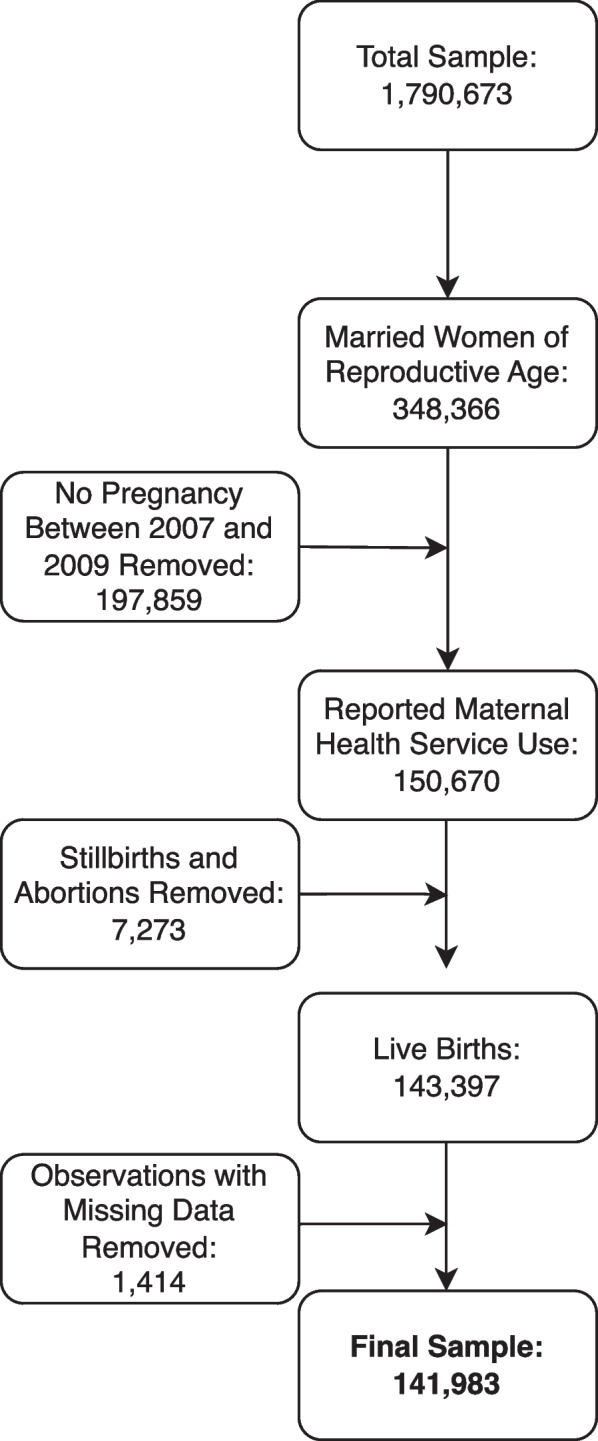
Sample data flow

A total of 150,670 women completed the survey at baseline. Women who reported stillbirths or abortions (7,273 women) were removed from the sample because there were missing data for all of the variables of interest for this study among the majority (over 75%) of women who had experienced stillbirth. An additional 1,414 women were removed as a result of missing demographic data. This left a final sample of 141,983 women. However, due to missing data on outcome questions, sample sizes vary for each of the outcomes included in the analysis. A data flow chart can be seen in Fig. 1.

### Measures

#### Outcomes

This study analyzed outcomes focused on ANC, skilled delivery, and PNC. All outcomes were included as dichotomous variables, coded as yes or no if the service of interest had been received. For ANC, the outcome of interest was whether women attended the minimum Indian government recommended three ANC visits during their pregnancy. Due to 5% of the sample having missing data regarding ANC visits, the sample size for this outcome is 135,563 women. If a woman reported she delivered at a facility or that her home delivery was assisted by a trained provider (doctor, midwife, or nurse), she was coded as having a skilled delivery. Due to missing responses regarding place of delivery, the sample size for this outcome is 141,865 women. To measure PNC, women were asked if they received PNC within 48 h of their delivery. For this question, 227 women reported they did not know and they were coded as missing. Due to missing data, the sample size for this analysis is 141,018 women.

#### Exposure

Survey participants were asked if they had any form of disability at the time of the survey. Their responses were then coded into eight categories based on the functions most impacted by their disability: mental, visual, hearing, speech, locomotor, multiple, other, or no disability. Symptoms were self-reported by the participants but coded into the categories by the survey enumerators. In this study, because of small samples for each of the disability categories, disability was changed to a dichotomous variable, coded as yes have a disability or no do not have a disability. Because of low prevalence of disability in the dataset, it was not possible to analyze the data using disability type. Some categories of disability such small numbers of participants once observations were dropped from the study any statistical testing could not be successfully attempted.

#### Covariates

A group of individual-level covariates was selected for inclusion in the model analyses. These variables were selected because they have significant relationships with disability and with the maternal healthcare use outcomes of interest. These included: the age of the mother; religion; social category (caste); education; marital status; and the number of living children at the time of the survey. A variable of first versus later order births was also created for later model stratification based on the number of living children reported. See Table 1 for specific categories within these covariates.

**Table 1:** Descriptive Statistics of Women Who Had a Live Birth, Rajasthan, 2010/11 AHS Survey Round

	**Number**	**Weighted Percentage**
**Total**	**141,983**	**100.00%**
**Disability**		
Yes	1793	1.23%
No	140,190	98.77%
**Disability Type (among those with a disability; *n* = 1793)**		
Mental	213	12.36%
Visual	384	20.83%
Hearing	105	5.96%
Speech	66	3.49%
Locomotor	874	48.62%
Multiple	131	7.65%
Other	20	1.10%
**Marital Status**		
Currently married	140,872	99.27%
Formerly Married	1,111	0.73%
**Religion**		
Hindu	126,728	88.08%
Muslim	13,138	10.43%
Other	2,117	1.49%
**Caste or Tribe**		
Other Caste	87,986	62.41%
Scheduled Caste	27,812	19.43%
Scheduled Tribe	26,185	18.17%
**Education**		
No Formal Education	82,977	56.96%
Up to Primary	26,668	18.50%
Middle	15,568	11.31%
Class 10 or Class 12	11,184	8.42%
Higher Ed	5,586	4.81%
**Age at Reported Birth**		
15–19	9,145	6.44%
20–34	122,721	86.43%
35 +	10,117	7.13%
**Mean Age**	25.74	
**Mean Parity**	2.83	
**Birth Order**		
First Order Birth	49,429	35.47%
Later Order Birth	92,554	64.53%
**Residence**		
Rural	122,354	79.77%
Urban	19,629	20.23%
**Overall Sex Ratio**^**a**^		
High	114,179	80.42%
Low	27,804	19.58%
**Population Served Per Facility**^**a**^		
3000–3499	24,316	17.13%
3500–3999	50,956	35.89%
4000–4499	27,959	19.69%
4500–4999	22,306	15.71%
5000 +	16,446	11.58%
**Human Development Index**^**a**^		
Low (0–0.549)	32,229	22.70%
Medium (0.55–0.699)	83,360	58.71%
High (0.7–0.799)	23,169	16.32%
Very High (0.8–1)	3,225	2.27%
**Percent of Villages < 200 Pop**^**a**^		
< 5%	61,474	43.30%
5–9.99%	54,479	38.37%
> = 10%	26,030	18.33%
**Minimum ANC Visits (3 +)**		
Yes	71,077	53.08%
No	64,486	46.92%
Total	135,563	100.00%
**Skilled Delivery**		
Yes	118,474	83.89%
No	23,391	16.11%
Total	141,865	100.00%
**PNC Within 48 Hours**		
Yes	107,200	76.45%
No	33,818	23.55%
Total	141,018	100.00%

^a^Unweighted percentage due to these variables being added to the dataset and not collected as part of the original survey

Economic development, healthcare infrastructure, and gender empowerment can all have an impact on maternal healthcare use and accessibility. Information on these variables was available at the district level from a variety of sources and was included in the analysis as controls. Economic development was measured via the Human Development Index (HDI) score for each district. Accessibility of healthcare in each district was measured via the average population served per medical institution in each district. This was calculated by taking the population of the district and dividing by the number of medical institutions in the district. Gender equality is included in the model as the population sex ratio, which includes all males and females of all ages in the district in the calculation. This ratio was analyzed based on guidance from the UN Population Fund [17]. Data for the three variables above was drawn from the 2008 Human Development Report [18]. The AHS did not include villages under 200 population in the sampling frame for data collection. This has the potential to skew the data, so the percentage of villages under 200 population in each district is included in the analysis to control for the impact of exclusion of these villages. This information came from the 2011 National Census [19]. Details regarding categories within these variables can be found in Table 1.

### Data analysis

We first described the full sample using descriptive analysis. Respondents were selected with unequal probability so sampling weights were needed and weighted percentages were used. We used chi-square tests to assess the significance of the relationship between disability and all outcomes across all covariates as all variables were categorical or count data. These analyses were used in combination with theory and other similar studies to determine the covariates that should be included in the models.

Logistic regression was used to determine the association between disability and each covariate with attending the minimum number of ANC visits, having a skilled delivery, and receiving PNC within 48 h of delivery. Three versions of the models were tested: a crude model that only includes disability as the primary exposure; a multivariate model including disability and all of the individual-level covariates described above; and a multilevel model including disability, all of the individual-level covariates, and all of the district-level covariates. These variables were selected based on the results of the bivariate analysis, theory, and variables included in similar studies.

Previous studies have shown that the order of birth (whether it is a woman’s first birth or a later birth) has an effect on maternal healthcare use [11]. Therefore, stratified models were created based on birth order, with separate models for the sample of women for whom the reported birth was their first birth and for the sample of women who were reporting on a later birth. There was a significantly larger proportion of WWD in rural areas than in urban areas. Place of residence defined as rural or urban is associated with use of maternal healthcare [11]. To account for this relationship, stratified models were developed for rural and urban women.

R squared values were used to determine how much of the variance in the outcomes could be attributed to disability and compared between models and the goodness of fit of the models.

## Results

### Description of the sample

Descriptive analyses were conducted on the sample of 141,983 ever married women who reported a live birth and completed all of the relevant demographic questions; findings are presented in Table 1. Of the women in this sample, 1,793 (1.2%) reported a disability. Locomotor disabilities were the most common disability category (48.6%). Among the women who completed the ANC questions, 53.1% reported attending three or more ANC visits during their pregnancy. The majority of women (83.9%) who completed the delivery questions reported a skilled delivery. Of the women who completed the PNC questions, 76.5% reported receiving PNC within 48 h of birth (Table 1).

### Bivariate analysis

In bivariate analyses, disability was significantly inversely associated with attending the minimum number of ANC visits, with a larger percentage of WWD reporting they had not attended the visits compared to women without disabilities. See Online Resource 1 for detailed results. There was no statistically significant relationship between disability and skilled delivery found. Results are presented in Online Resource 2. Receiving PNC within 48 h of delivery was reported by a significantly smaller percentage of WWD than reported not receiving PNC within 48 h. These results are presented in Online Resource 3.

### Regression models – minimum ANC visits

Table 2 presents the results of the regression analysis regarding attending three or more ANC visits. In the crude model, WWD had lower odds of reporting minimum ANC visits (OR = 0.72; CI:0.65,0.79). In the multivariate model, disability was still significant, with WWD having lower odds of reporting minimum ANC visits (OR = 0.86; CI:0.78,0.95). WWD still reported lower odds of attending the minimum ANC visits (OR = 0.84; CI:0.76,0.92) in the multilevel model.

**Table 2:** Logistic Regression Models, Minimum Antenatal Care Visits

	*Unadjusted Model*	*Adjusted Model: Individual*	*Adjusted Model: Individual and District*
	*N* = 135,563	*N* = 135,563	*N* = 135,563
*Predictor Variable*			
Disability (no)			
Yes	0.72[0.65;0.79]***	0.86[0.78;0.95]**	0.84[0.76;0.92]***
*Individual Covariates*			
Age at Birth (20–34)			
15–19		0.73[0.70;0.76]***	0.76[0.72;0.79]***
30–34		1.09[1.04;1.14]***	1.07[1.02;1.12]**
Marital Status (Currently Married)			
Formerly Married		0.94[0.83;1.06]	0.89[0.76;1.01]
Religion (Hindu)			
Muslim		1.28[1.23;1.33]***	1.13[1.08;1.18]***
Other		1.36[1.24;1.50]***	1.32[1.19;1.47]***
Social Group (Other Caste)			
Scheduled Caste		0.82[0.80;0.85]***	0.76[0.74;0.79]***
Scheduled Tribe		1.00[1.24;1.50]	0.99[0.96;1.02]
Highest Education (No education)			
Up to Primary		1.50[1.46;1.55]***	1.45[1.41;1.50]***
Middle		1.84[1.77;1.91]***	1.79[1.73;1.86]***
Class 10 or 12		2.19[2.10;2.29]***	2.08[1.99;2.18]***
Higher Education		3.24[3.03;3.46]***	2.67[2.49;2.86]***
Parity		0.90[0.89;0.90]***	0.90[0.90;0.91]***
*District Covariates*			
Residence (Rural)			
Urban			1.69[1.63;1.75]***
Sex Ratio Categories (High)			
Low			0.63[0.60;0.65]***
Population Served per Medical Institution (3000–3499)			
3500–3999			0.88[0.84;0.92]***
4000–4499			1.24[1.17;1.31]***
4500–4999			1.12[1.06;1.18]***
5000 +			2.32[2.18;2.47]***
HDI Score Category (Low)			
Medium (0.55–0.699)			0.73[0.70;0.77]***
High (0.7–0.799)			0.58[0.55;0.62]***
Very High (0.8–1)			0.74[0.66;0.82]***
% Villages < 200 Pop (< 5%)			
5–9.99%			1.38[1.34;1.42]***
> = 10%			1.58[1.52;1.64]***

^*^p > 0.05

^**^p > 0.01

^***^p > 0.001

Results of the birth order and residence stratified models can be found in Table 3. Birth order did not have an impact on attending ANC visits, with WWD experiencing their first birth and a later birth both reporting lower odds of minimum ANC visits than women without disabilities in all models. Rural WWD had significantly lower odds of reporting attending the minimum ANC visits in the crude (OR = 0.75; CI:0.67,0.83), multivariate (OR = 0.86; CI:0.77,0.95), and multilevel (OR = 0.84; CI:0.76,0.93) models. There was no significant relationship between disability and minimum ANC visits among urban women in the multivariate or multilevel models. R squared values in all of the crude models were close to 0, showing a limited amount of the variability in use of ANC could be attributed directly to disability.

**Table 3:** Birth Order and Residence Stratified Logistic Regression Models, Minimum Antenatal Care Visits

	*Unadjusted*	*Adjusted: Individual*	*Adjusted: Individual and District*	*Unadjusted*	*Adjusted: Individual*	*Adjusted: Individual and District*
	**First Order Birth**			**Later Order Birth**		
	*N* = 47,729	*N* = 47,729	*N* = 47,729	*N* = 87,834	*N* = 87,834	*N* = 87,834
Disability						
Yes	0.66[0.56;0.79]***	0.78[0.65;0.94]**	0.78[0.65;0.94]**	0.76[0.67;0.85]***	0.87[0.77;0.97]*	0.84[0.75;0.95]**
	**Rural**			**Urban**		
	*N* = 116,344	*N* = 116,344	*N* = 116,344	*N* = 19,219	*N* = 19,219	*N* = 19,219
Disability						
Yes	0.75[0.67;0.83]***	0.86[0.77;0.95]**	0.84[0.76;0.93]**	0.66[0.49;0.91] **	0.77[0.56;1.06]	0.76[0.55;1.05]

^*^p > 0.05

^**^p > 0.01

^***^p > 0.001

### Regression models – skilled delivery

There was no significant relationship between disability and skilled delivery in the crude, multivariate, or multilevel unstratified models. These results are presented in Online Resource 4. Results of the stratified models are found in Online Resource 5. WWD were more likely to report skilled delivery only in the multilevel, first birth stratified model (OR = 1.42; CI:1.04,1.93). There was no significant relationship between skilled delivery and disability in the later order birth, rural, or urban stratified models. Again, R squared values in all of the crude models were close to 0, showing a limited amount of the variability in use of skilled delivery could be attributed directly to disability.

### Regression models – postnatal care within 48 h

Results from the unstratified models of PNC within 48 h can be found in Online Resource 6. There was no significant relationship between disability and receiving PNC in the multivariate or multilevel unstratified models. However, the relationship was significant in the crude, unstratified model, with WWD reporting lower odds of receiving PNC (OR = 0.87; CI:0.78,0.96). WWD were more likely to report receiving PNC in the first order birth stratified, multivariate (OR = 1.45; CI:1.12,1.88) and multilevel (OR = 1.47; CI:1.13,1.91) models, but not in the crude model. In the later order birth stratified models, disability was only significant in the crude model, with WWD reporting lower odds of receiving PNC (OR = 0.83; CI:0.73,0.93). In the rural stratified model, WWD had lower odds of PNC in the crude model (OR = 0.89; CI:0.79,0.99), but disability was not significant in the multivariate or multilevel models. Disability did not have a significant impact on receiving PNC in any of the urban stratified models. These results are located in Online Resource 7. As with the previous outcomes, R squared values in all of the PNC crude models were close to 0, little of the variability in use of PNC could be attributed to disability.

## Discussion

There is a lack of quantitative research regarding maternal healthcare use among WWD in India. The AHS data from 2011 is the most recent dataset containing both disability and maternal health care use in a single survey and is therefore the most relevant data to use for this study. This study adds to the literature regarding maternal health of WWD in India in general and Rajasthan specifically, filling important gaps in maternal health research in the region. There are several key takeaways from this study. WWD have lower odds of attending at least three ANC visits than women without disabilities in Rajasthan. This is of concern because ANC is an essential part of maternal healthcare. Women who receive ANC have better birth outcomes, complications can be identified earlier, and they are more likely to deliver at a facility with a skilled provider [20]. For example, a study in Taiwan found WWD used fewer ANC services and had higher risk of preterm birth [21]. Because of this, all women should have access to ANC, regardless of their disability status [13]. In some models, WWD also had lower odds of receiving PNC within 48 h of birth. No evidence could be found that WWD who had facility births left the facility before 48 h had elapsed at higher rates than women without disabilities and there were no significant differences found in hospital versus facility births, so this should not impact the use of PNC within 48 h of the birth. The finding of lower PNC use among WWD parallels a study in Vietnam that women with physical disabilities attended ANC appointments, but not PNC because of the quality of care provided during the ANC and delivery [22]. PNC is vital for identifying pregnancy related injuries, post-delivery complications like postpartum hemorrhage and infection, and newborn health issues [23]. These causes of maternal morbidity and mortality occur in the first couple of days after birth, and having access to PNC in that time frame is vital for addressing these issues [23].

Some of the findings of this study align with the results of studies assessing use of maternal healthcare among WWD and some do not. One quantitative study has assessed maternal healthcare and disability in South India. This specific study used a case–control design, matching 247 WWD with 324 age-matched controls aged 15–45 years. The authors did not find a statistically significant difference in use of ANC, hospital delivery, Cesarean section, or pregnancy outcomes [24]. The difference in findings could be because of the difference in sample size, location of the study, or study design. This study takes place in South India, where other studies have found women have higher maternal healthcare use overall compared to women in North India [25]. In addition, the lack of significant associations between disability and maternal healthcare use is corroborated by a similar study using the Demographic and Health Surveys in Pakistan [26].

The findings of this study could prove useful when addressing issues that can reduce access to maternal healthcare for WWD. For example, there is a feeling among WWD that providers are not receiving adequate training regarding the unique concerns of WWD, and this creates stigma regarding pregnancy among maternal providers [27, 28]. Studies interviewing obstetric care providers have found evidence of this lack of training in a number of locations [28–30]. Many maternal providers in India are trained or employed directly by the government [31]. Therefore, knowing that there are differences in maternal health needs for WWD and that there are disparities in use of maternity care in India, the government could implement training regarding maternal health and disability among providers.

According to Census data, WWD are more likely to be living in rural areas in India [32]. The National Rural Health Mission specifically works in this region, and should focus on accessibility to ensure integration of WWD into their maternal health programming [33]. The Rights of Persons with Disabilities Act also guarantees maternal healthcare for WWD [34]. Up to this point, implementation of the act has been extremely limited [35], and acting on the regulations related to maternal health have been especially difficult because of a lack of data and research regarding maternal healthcare and disability. The Indian government and the government of Rajasthan could use the findings of this study to aid them in implementing this law.

## Conclusions

### Further research

There are a number of additional studies that need to be conducted to determine the impact of disability on maternal healthcare use in India. A first step is to analyze the data from other states included in the AHS to determine if these findings can be replicated. Also, including disability as a variable in other, nationally representative maternal health surveys like the NFHS would allow for studies to determine if associations between disability and maternal healthcare are found at the national level.

The sample of WWD in this study was too small to consider differences in maternal healthcare use by disability type. A number of studies have been conducted in other countries looking at maternal healthcare usage and quality of care received for women in specific disability type categories. In general, these studies have found increased disparities in maternal healthcare use among women with cognitive disabilities in particular [36–38]. Additional studies will be needed in India to determine if there are differences in maternal healthcare use by women with different types of disabilities.

### Limitations

Because this dataset consists of retrospective data, there is a potential for recall bias in this study [39]. The data were collected via face-to-face interviews, creating the potential for interviewer bias or social desirability bias [40]. The lack of inclusion of questions regarding the timing of disability means it is impossible to determine if the disability or births occurred first. This is particularly important for maternal health research, as maternal morbidities can cause long-term disabilities. Having a disability as a result of maternal morbidity could impact later maternal health use [41]. Because of the lack of disability timing data, this study can only determine associations between disability and maternal healthcare use, not causal relationships. There are also some differential demographics between women who did and did not complete outcome questions, which could have a biasing effect on the study analysis. R-squared results for the disability-only models are zero, which shows statistically significant association between disability and the outcomes of this study are limited in how meaningful they are. It is possible these significant associations have been found because of the large size of the sample. Additional studies would be needed to determine if a meaningful association can be identified.

### Practice implications

This study is the just the beginning of what should be a great deal of research regarding maternal health of WWD in India. Based on this study, there are differences in maternal healthcare usage among WWD. Researchers need to determine if these differences also cause disparities in maternal and infant health outcomes. Additional analysis needs to be conducted to determine if these results can be replicated in other states of India. States in India have implemented the recommendations of previous accessibility policies in different ways and to varied success. This leads to the potential that maternal health services are more or less accessible in other states than they are in Rajasthan [42].

Maternal health programming needs to be made accessible to WWD. Policies regarding equity in maternal healthcare access need to be passed and enforced to ensure these rights are respected within society and the healthcare system. This should be a focus of not only the fields of obstetrics and public health, but also providers in fields focused on PWD, such as rehabilitation. These providers who already have expertise in working with patients with disabilities could prove invaluable when creating a more accessible maternal health system.

Other surveys collecting data on maternal and child health need to include disability as a variable. Ideally, this would be done using questionnaires developed by experts in disability and measured in a way that can be compared across countries. All of these things will assist the field of public health to develop a more well-rounded understanding of how WWD use and navigate the maternal healthcare system to inform improved accessibility to these services for all WWD.

### Supplementary Information

**Additional file 1: Online Resource 1.** Bivariate Analysis of Covariates and Minimum Antenatal Care Visits. **Online Resource 2.** Bivariate Analysis of Covariates and Skilled Delivery. **Online Resource 3.** Bivariate Analysis of Covariates and Postnatal Care Within 48 Hours. **Online Resource 4.** Logistic Regression Models, Skilled Delivery. **Online Resource 5.** Birth Order and Residence Stratified Logistic Regression Models, Skilled Delivery. **Online Resource 6.** Logistic Regression Models, Postnatal Care within 48 Hours. **Online Resource 7.** Birth Order and Residence Stratified Logistic Regression Models, Postnatal Care within 48 Hours.

## Data Availability

The data that support the findings of this study are openly available in the Open Government Data Platform India at https://data.gov.in/catalog/annual-health-survey-woman-schedule?filters%5Bfield_catalog_reference%5D=1101141&format=json&offset=0&limit=6&sort%5Bcreated%5D=desc.

## Abbreviations

AHS: Annual Health Survey
ANC: Antenatal care
HDI: Human Development Index
PNC: Postnatal care
PWD: People with disabilities
WWD: Women with disabilities
