# Supplementary material for: Maternal healthcare use by women with disabilities in Rajasthan, India: a secondary analysis of the Annual Health Survey
Source: Matern Health Neonatol Perinatol. 2023 Sep 4;9:11. doi: 10.1186/s40748-023-00165-1 (PMC10476301; doi:10.1186/s40748-023-00165-1)
Supplement: Supplementary file 1 — Additional file 1: Online Resource 1. Bivariate Analysis of Covariates and Minimum Antenatal Care Visits. Online Resource 2. Bivariate Analysis of Covariates and Skilled Delivery. Online Resource 3. Bivariate Analysis of Covariates and Postnatal Care Within 48 Hours. Online Resource 4. Logistic Regression Models, Skilled Delivery. Online Resource 5. Birth Order and Residence Stratified Logistic Regression Models, Skilled Delivery. Online Resource 6. Logistic Regression Models, Postnatal Care within 48 Hours. Online Resource 7. Birth Order and Residence Stratified Logistic Regression Models, Postnatal Care within 48 Hours. [file 40748_2023_165_MOESM1_ESM.docx]

Online Resource 1: Bivariate Analysis of Covariates and Minimum Antenatal Care Visits

|  | **3+ ANC Visits** | | **<3 ANC Visits** | | **Total** | | **Chi Square** |
| --- | --- | --- | --- | --- | --- | --- | --- |
|  | **N** | **%** | **N** | **%** | **N** | **%** |  |
| **Disability** |  |  |  |  |  |  | 46.1718*** |
| Yes | 746 | 1.05% | 941 | 1.46% | 1687 | 1.24% |  |
| No | 70331 | 98.95% | 63545 | 98.54% | 133876 | 98.76% |  |
| Total | 71077 | 100.00% | 64486 | 100.00% | 135563 | 100.00% |  |
| **Marital Status** |  |  |  |  |  |  | 3.9945* |
| Currently married | 70548 | 99.26% | 63944 | 99.16% | 134492 | 99.21% |  |
| Formerly Married | 529 | 0.74% | 542 | 0.84% | 1071 | 0.79% |  |
| Total | 71077 | 100.00% | 64486 | 100.00% | 135563 | 100.00% |  |
| **Religion** |  |  |  |  |  |  | 390.0347*** |
| Hindu | 62500 | 87.93% | 58600 | 90.87% | 121100 | 89.33% |  |
| Muslim | 7156 | 10.07% | 5235 | 8.12% | 12391 | 9.14% |  |
| Other | 1421 | 2.00% | 651 | 1.01% | 2072 | 1.53% |  |
| Total | 71077 | 100.00% | 64486 | 100.00% | 135563 | 100.00% |  |
| **Caste or Tribe** |  |  |  |  |  |  | 1200*** |
| Other Caste | 46821 | 65.87% | 37362 | 57.94% | 84183 | 62.10% |  |
| Scheduled Caste | 11527 | 16.22% | 14961 | 23.20% | 26488 | 19.54% |  |
| Scheduled Tribe | 12729 | 17.91% | 12163 | 18.86% | 24892 | 18.36% |  |
| Total | 71077 | 100.00% | 64486 | 100.00% | 135563 | 100.00% |  |
| **Education** |  |  |  |  |  |  | 5300*** |
| No Formal Education | 34889 | 49.09% | 43231 | 67.04% | 78120 | 57.63% |  |
| Up to Primary | 14848 | 20.89% | 10867 | 16.85% | 25715 | 18.97% |  |
| Middle | 9618 | 13.53% | 5589 | 8.67% | 15207 | 11.22% |  |
| Class 10 or Class 12 | 7472 | 10.51% | 3519 | 5.46% | 10991 | 8.11% |  |
| Higher Ed | 4250 | 5.98% | 1280 | 1.98% | 5530 | 4.08% |  |
| Total | 71077 | 100.00% | 64486 | 100.00% | 135563 | 100.00% |  |
| **Age at Reported Birth** |  |  |  |  |  |  | 561.1897*** |
| 15-19 | 4451 | 6.26% | 4477 | 6.94% | 8928 | 6.59% |  |
| 20-24 | 31638 | 44.51% | 26026 | 40.36% | 57664 | 42.54% |  |
| 25-29 | 22341 | 31.43% | 19908 | 30.87% | 42249 | 31.17% |  |
| 30-34 | 8615 | 12.12% | 8700 | 13.49% | 17315 | 12.77% |  |
| 35-39 | 2693 | 3.79% | 3534 | 5.48% | 2693 | 3.79% |  |
| 40-44 | 912 | 1.28% | 1280 | 1.98% | 2192 | 1.62% |  |
| 45-49 | 427 | 0.60% | 561 | 0.87% | 988 | 0.73% |  |
| Total | 71077 | 100.00% | 64486 | 100.00% | 135563 | 100.00% |  |
| **Birth Order** |  |  |  |  |  |  | 1100*** |
| First Order Birth | 27939 | 39.31% | 19790 | 30.69% | 47729 | 35.21% |  |
| Later Order Birth | 43138 | 60.69% | 44696 | 69.31% | 87834 | 64.79% |  |
| Total | 71077 | 100.00% | 64486 | 100.00% | 135563 | 100.00% |  |
| **Residence** |  |  |  |  |  |  | 3100*** |
| Rural | 57409 | 80.77% | 58935 | 91.39% | 116344 | 85.82% |  |
| Urban | 13668 | 19.23% | 5551 | 8.61% | 19219 | 14.18% |  |
| Total | 71077 | 100.00% | 64486 | 100.00% | 135563 | 100.00% |  |
| **Overall Sex Ratio** |  |  |  |  |  |  | 230.8259*** |
| High | 58251 | 81.95% | 50734 | 78.67% | 108985 | 80.39% |  |
| Low | 12826 | 18.05% | 13752 | 21.33% | 26578 | 19.61% |  |
| Total | 71077 | 100.00% | 64486 | 100.00% | 135563 | 100.00% |  |
| **Pop Served Per Facility** |  |  |  |  |  |  | 2500*** |
| 3000-3499 | 10974 | 15.44% | 11757 | 18.23% | 22731 | 16.77% |  |
| 3500-3999 | 23253 | 32.72% | 25190 | 39.06% | 48443 | 35.73% |  |
| 4000-4499 | 15699 | 22.09% | 11418 | 17.71% | 27117 | 20.00% |  |
| 4500-4999 | 10314 | 14.51% | 10979 | 17.03% | 21293 | 15.71% |  |
| 5000+ | 10837 | 15.25% | 5142 | 7.97% | 15979 | 11.79% |  |
| Total | 71077 | 100.00% | 64486 | 100.00% | 135563 | 100.00% |  |
| **Human Development Index** |  |  |  |  |  |  | 903.6488*** |
| Low (0-0.549) | 14681 | 20.66% | 16086 | 24.94% | 30767 | 22.70% |  |
| Medium (0.55-0.699) | 41037 | 57.74% | 38235 | 59.29% | 79272 | 58.48% |  |
| High (0.7-0.799) | 13433 | 18.90% | 8949 | 13.88% | 22382 | 16.51% |  |
| Very High (0.8-1) | 1926 | 2.71% | 1216 | 1.89% | 3142 | 2.32% |  |
| Total | 71077 | 100.00% | 64486 | 100.00% | 135563 | 100.00% |  |
| **Percent of Villages <200 Pop** |  |  |  |  |  |  | 542.1950*** |
| <5% | 28381 | 39.93% | 29335 | 45.49% | 57716 | 42.58% |  |
| 5-9.99% | 28248 | 39.74% | 24527 | 38.03% | 52775 | 38.93% |  |
| >=10% | 14448 | 20.33% | 10624 | 16.47% | 25072 | 18.49% |  |
| Total | 71077 | 100.00% | 64486 | 100.00% | 135563 | 100.00% |  |
| * p<0.05, **p<0.01, ***p<0.001 | | | | | | | |

Online Resource 2: Bivariate Analysis of Covariates and Skilled Delivery

|  | **Skilled Delivery** | | **Unskilled Delivery** | | **Total** | | **Chi Square** |
| --- | --- | --- | --- | --- | --- | --- | --- |
|  | **N** | **%** | **N** | **%** | **N** | **%** |  |
| **Disability** |  |  |  |  |  |  | 3.7828 |
| Yes | 1467 | 1.24% | 326 | 1.39% | 1793 | 1.26% |  |
| No | 117007 | 98.76% | 23065 | 98.61% | 140072 | 98.74% |  |
| Total | 118474 | 100.00% | 23391 | 100.00% | 141865 | 100.00% |  |
| **Marital Status** |  |  |  |  |  |  | 12.6487*** |
| Currently married | 117590 | 99.25% | 23164 | 99.03% | 140754 | 99.22% |  |
| Formerly Married | 884 | 0.75% | 227 | 0.97% | 1111 | 0.78% |  |
| Total | 118474 | 100.00% | 23391 | 100.00% | 141865 | 100.00% |  |
| **Religion** |  |  |  |  |  |  | 75.8015*** |
| Hindu | 105635 | 89.16% | 20998 | 89.77% | 126633 | 89.26% |  |
| Muslim | 10928 | 9.22% | 2192 | 9.37% | 13120 | 9.25% |  |
| Other | 1911 | 1.61% | 201 | 0.86% | 2112 | 1.49% |  |
| Total | 118474 | 100.00% | 23391 | 100.00% | 141865 | 100.00% |  |
| **Caste or Tribe** |  |  |  |  |  |  | 791.5987*** |
| Other Caste | 74936 | 63.25% | 12970 | 55.45% | 87906 | 61.96% |  |
| Scheduled Caste | 21702 | 18.32% | 6099 | 26.07% | 27801 | 19.60% |  |
| Scheduled Tribe | 21836 | 18.43% | 4322 | 18.48% | 26158 | 18.44% |  |
| Total | 118474 | 100.00% | 23391 | 100.00% | 141865 | 100.00% |  |
| **Education** |  |  |  |  |  |  | 4500*** |
| No Formal Education | 64887 | 54.77% | 18023 | 77.05% | 82910 | 58.44% |  |
| Up to Primary | 23340 | 19.70% | 3302 | 14.12% | 26642 | 18.78% |  |
| Middle | 14278 | 12.05% | 1279 | 5.47% | 15557 | 10.97% |  |
| Class 10 or Class 12 | 10550 | 8.90% | 626 | 2.68% | 11176 | 7.88% |  |
| Higher Ed | 5419 | 4.57% | 161 | 0.69% | 5580 | 3.93% |  |
| Total | 118474 | 100.00% | 23391 | 100.00% | 141865 | 100.00% |  |
| **Age at Reported Birth** |  |  |  |  |  |  | 751.9984*** |
| 15-19 | 7806 | 6.59% | 1326 | 5.67% | 9132 | 6.44% |  |
| 20-24 | 51329 | 43.33% | 8527 | 36.45% | 59856 | 42.19% |  |
| 25-29 | 36834 | 31.09% | 7506 | 32.09% | 44340 | 31.26% |  |
| 30-34 | 14741 | 12.44% | 3686 | 15.76% | 18427 | 12.99% |  |
| 35-39 | 5151 | 4.35% | 1532 | 6.55% | 6683 | 4.71% |  |
| 40-44 | 1799 | 1.52% | 586 | 2.51% | 2385 | 1.68% |  |
| 45-49 | 814 | 0.69% | 228 | 0.97% | 1042 | 0.73% |  |
| Total | 118474 | 100.00% | 23391 | 100.00% | 141865 | 100.00% |  |
| **Birth Order** |  |  |  |  |  |  | 2200**** |
| First Order Birth | 44359 | 37.44% | 5031 | 21.51% | 49390 | 34.81% |  |
| Later Order Birth | 74115 | 62.56% | 18360 | 78.49% | 92475 | 65.19% |  |
| Total | 118474 | 100.00% | 23391 | 100.00% | 141865 | 100.00% |  |
| **Residence** |  |  |  |  |  |  | 1700*** |
| Rural | 100112 | 84.50% | 22149 | 94.69% | 122261 | 86.18% |  |
| Urban | 18362 | 15.50% | 1242 | 5.31% | 19604 | 13.82% |  |
| Total | 118474 | 100.00% | 23391 | 100.00% | 141865 | 100.00% |  |
| **Overall Sex Ratio** |  |  |  |  |  |  | 148.0182*** |
| High | 94614 | 79.86% | 19488 | 83.31% | 114102 | 80.43% |  |
| Low | 23860 | 20.14% | 3903 | 16.69% | 27763 | 19.57% |  |
| Total | 118474 | 100.00% | 23391 | 100.00% | 141865 | 100.00% |  |
| **Pop Served Per Facility** |  |  |  |  |  |  | 2000*** |
| 3000-3499 | 18300 | 17.13% | 6001 | 25.66% | 24301 | 17.13% |  |
| 3500-3999 | 42041 | 35.49% | 8891 | 38.01% | 50932 | 35.90% |  |
| 4000-4499 | 24146 | 20.38% | 3775 | 16.14% | 27921 | 19.68% |  |
| 4500-4999 | 19299 | 16.29% | 2995 | 12.80% | 22294 | 15.71% |  |
| 5000+ | 14688 | 12.40% | 1729 | 7.39% | 1647 | 11.57% |  |
| Total | 118474 | 100.00% | 23391 | 100.00% | 141865 | 100.00% |  |
| **Human Development Index** |  |  |  |  |  |  | 1000*** |
| Low (0-0.549) | 25719 | 21.71% | 6498 | 27.78% | 32217 | 22.71% |  |
| Medium (0.55-0.699) | 69226 | 58.43% | 14084 | 60.21% | 83310 | 58.72% |  |
| High (0.7-0.799) | 20521 | 17.32% | 2604 | 11.13% | 23125 | 16.30% |  |
| Very High (0.8-1) | 3008 | 2.54% | 205 | 0.88% | 3213 | 2.26% |  |
| Total | 118474 | 100.00% | 23391 | 100.00% | 141865 | 100.00% |  |
| **Percent of Villages <200 Pop** |  |  |  |  |  |  | 897.5765*** |
| <5% | 49301 | 41.61% | 12123 | 51.83% | 61424 | 43.30% |  |
| 5-9.99% | 46403 | 39.17% | 8029 | 34.33% | 54432 | 38.37% |  |
| >=10% | 22770 | 19.22% | 3239 | 13.85% | 26009 | 18.33% |  |
| Total | 118474 | 100.00% | 23391 | 100.00% | 141865 | 100.00% |  |
| * p<0.05, **p<0.01, ***p<0.001 | | | | | | | |

Online Resource 3: Bivariate Analysis of Covariates and Postnatal Care Within 48 Hours

|  | **PNC within 48 hrs** | | **No PNC within 48 hrs** | | **Total** | | **Chi Square** |
| --- | --- | --- | --- | --- | --- | --- | --- |
|  | **N** | **%** | **N** | **%** | **N** | **%** |  |
| **Disability** |  |  |  |  |  |  | 6.9333** |
| Yes | 1306 | 1.22% | 474 | 1.40% | 1780 | 1.26% |  |
| No | 105894 | 98.78% | 33344 | 98.60% | 139238 | 98.74% |  |
| Total | 107200 | 100.00% | 33818 | 100.00% | 141018 | 100.00% |  |
| **Marital Status** |  |  |  |  |  |  | 1.7101 |
| Currently married | 106386 | 99.24% | 33537 | 99.17% | 139923 | 99.22% |  |
| Formerly Married | 814 | 0.76% | 281 | 0.83% | 1095 | 0.78% |  |
| Total | 107200 | 100.00% | 33818 | 100.00% | 141018 | 100.00% |  |
| **Religion** |  |  |  |  |  |  | 75.5652*** |
| Hindu | 95522 | 89.11% | 30339 | 89.71% | 125861 | 89.25% |  |
| Muslim | 9904 | 9.24% | 3142 | 9.29% | 13046 | 9.25% |  |
| Other | 1774 | 1.65% | 337 | 1.00% | 2111 | 1.50% |  |
| Total | 107200 | 100.00% | 33818 | 100.00% | 141018 | 100.00% |  |
| **Caste or Tribe** |  |  |  |  |  |  | 433.1863*** |
| Other Caste | 67869 | 63.31% | 19542 | 57.79% | 87411 | 61.99% |  |
| Scheduled Caste | 19751 | 18.42% | 7843 | 23.19% | 27594 | 19.57% |  |
| Scheduled Tribe | 19580 | 18.26% | 6433 | 19.02% | 26013 | 18.45% |  |
| Total | 107200 | 100.00% | 33818 | 100.00% | 141018 | 100.00% |  |
| **Education** |  |  |  |  |  |  | 4400*** |
| No Formal Education | 57804 | 53.92% | 24553 | 72.60% | 82357 | 58.40% |  |
| Up to Primary | 21154 | 19.73% | 5352 | 15.83% | 26506 | 18.80% |  |
| Middle | 13181 | 12.30% | 2297 | 6.79% | 15478 | 10.98% |  |
| Class 10 or Class 12 | 9886 | 9.22% | 1233 | 3.65% | 11119 | 7.88% |  |
| Higher Ed | 5175 | 4.83% | 383 | 1.13% | 5558 | 3.94% |  |
| Total | 107200 | 100.00% | 33818 | 100.00% | 141018 | 100.00% |  |
| **Age at Reported Birth** |  |  |  |  |  |  | 611.3347*** |
| 15-19 | 7063 | 6.59% | 2021 | 5.98% | 9084 | 6.44% |  |
| 20-24 | 46768 | 43.63% | 12833 | 37.95% | 59601 | 42.26% |  |
| 25-29 | 33230 | 31.00% | 10832 | 32.03% | 44062 | 31.25% |  |
| 30-34 | 13241 | 12.35% | 5065 | 14.98% | 18306 | 12.98% |  |
| 35-39 | 4582 | 4.27% | 2037 | 6.02% | 6619 | 4.69% |  |
| 40-44 | 1605 | 1.50% | 739 | 2.19% | 2344 | 1.66% |  |
| 45-49 | 711 | 0.66% | 291 | 0.86% | 1002 | 0.71% |  |
| Total | 107200 | 100.00% | 33818 | 100.00% | 141018 | 100.00% |  |
| **Birth Order** |  |  |  |  |  |  | 2200*** |
| First Order Birth | 40857 | 38.11% | 8225 | 24.32% | 49082 | 34.81% |  |
| Later Order Birth | 66343 | 61.68% | 25593 | 75.68% | 91936 | 65.19% |  |
| Total | 107200 | 100.00% | 33818 | 100.00% | 141018 | 100.00% |  |
| **Residence** |  |  |  |  |  |  | 1500*** |
| Rural | 90215 | 84.16% | 31257 | 92.43% | 121472 | 86.14% |  |
| Urban | 16985 | 15.84% | 2561 | 7.57% | 19546 | 13.86% |  |
| Total | 107200 | 100.00% | 33818 | 100.00% | 141018 | 100.00% |  |
| **Overall Sex Ratio** |  |  |  |  |  |  | 57.8617*** |
| High | 85794 | 80.03% | 27701 | 81.91% | 113495 | 80.48% |  |
| Low | 21406 | 19.97% | 6117 | 18.09% | 27523 | 19.52% |  |
| Total | 107200 | 100.00% | 33818 | 100.00% | 141018 | 100.00% |  |
| **Pop Served Per Facility** |  |  |  |  |  |  | 1900*** |
| 3000-3499 | 16032 | 14.96% | 8129 | 24.04% | 24161 | 17.13% |  |
| 3500-3999 | 38219 | 35.65% | 12430 | 36.76% | 50649 | 35.92% |  |
| 4000-4499 | 22448 | 20.94% | 5317 | 15.72% | 27765 | 19.69% |  |
| 4500-4999 | 17170 | 16.02% | 4855 | 14.36% | 22025 | 15.62% |  |
| 5000+ | 13331 | 12.44% | 3087 | 9.13% | 16418 | 11.64% |  |
| Total | 107200 | 100.00% | 33818 | 100.00% | 141018 | 100.00% |  |
| **Human Development Index** |  |  |  |  |  |  | 673.1437*** |
| Low (0-0.549) | 23257 | 21.69% | 8691 | 25.70% | 31948 | 22.66% |  |
| Medium (0.55-0.699) | 62392 | 58.20% | 20312 | 60.06% | 82704 | 58.65% |  |
| High (0.7-0.799) | 18876 | 17.61% | 4265 | 12.61% | 23141 | 16.41% |  |
| Very High (0.8-1) | 2675 | 2.50% | 550 | 1.63% | 3225 | 2.29% |  |
| Total | 107200 | 100.00% | 33818 | 100.00% | 141018 | 100.00% |  |
| **Percent of Villages <200 Pop** |  |  |  |  |  |  | 1000*** |
| <5% | 43942 | 40.99% | 17239 | 50.98% | 61181 | 43.39% |  |
| 5-9.99% | 42810 | 39.93% | 11262 | 33.30% | 54072 | 38.34% |  |
| >=10% | 20448 | 19.07% | 5317 | 15.72% | 25765 | 18.27% |  |
| Total | 107200 | 100.00% | 33818 | 100.00% | 141018 | 100.00% |  |
| * p<0.05, **p<0.01, ***p<0.001 | | | | | | | |

Online Resource 4: Logistic Regression Models, Skilled Delivery

|  | *Unadjusted Model* | *Adjusted Model: Individual* | *Adjusted Model: Individual and District* |
| --- | --- | --- | --- |
|  | N = 141865 | N = 141865 | N = 141865 |
| *Predictor Variable* |  |  |  |
| Disability (no) |  |  |  |
| Yes | 0.89 [0.79; 1.00] | 1.11 [0.98; 1.25] | 1.12 [0.99; 1.27] |
| *Individual Covariates* |  |  |  |
| Age at Birth (20-34) |  |  |  |
| 15-19 |  | 0.88 [0.82; 0.93]*** | 0.86 [0.1; 0.91]*** |
| 30-34 |  | 1.21 [1.14; 1.28]*** | 1.22 [1.15; 1.29]*** |
| Marital Status (Currently Married) |  |  |  |
| Formerly Married |  | 0.81 [0.70; 0.94]** | 0.77 [0.66; 0.90]** |
| Religion (Hindu) |  |  |  |
| Muslim |  | 0.99 [0.94; 1.04] | 0.89 [0.84; 0.94]*** |
| Other |  | 1.07 [0.92; 1.24] | 0.68 [0.58; 0.80]*** |
| Social Group (Other Caste) |  |  |  |
| Scheduled Caste |  | 0.83 [0.80; 0.87]*** | 0.76 [0.73; 0.79]*** |
| Scheduled Tribe |  | 1.04 [1.00; 1.09]* | 0.98 [0.94; 1.02] |
| Highest Education (No education) |  |  |  |
| Up to Primary |  | 1.70 [1.63; 1.77]*** | 1.64 [1.57; 1.71]*** |
| Middle |  | 2.58 [2.43; 2.74]*** | 2.36 [2.22; 2.51]*** |
| Class 10 or 12 |  | 3.75 [3.45; 4.08]*** | 3.29 [3.02; 3.58]*** |
| Higher Education |  | 7.08 [6.04; 8.30]*** | 5.30 [4.52; 6.23]*** |
| Parity |  | 0.87 [0.87; 0.88]*** | 0.88 [0.87; 0.88]*** |
| *District Covariates* |  |  |  |
| Residence (Rural) |  |  |  |
| Urban |  |  | 2.09 [1.97; 2.23]*** |
| Sex Ratio Categories (High) |  |  |  |
| Low |  |  | 0.98 [0.94; 1.03] |
| Population Served per Medical Institution (3000-3499) |  |  |  |
| 3500-3999 |  |  | 2.13 [2.00; 2.27]*** |
| 4000-4499 |  |  | 2.27 [2.11; 2.43]*** |
| 4500-4999 |  |  | 2.73 [2.55; 2.93]*** |
| 5000+ |  |  | 3.50 [3.21; 3.82]*** |
| HDI Score Category (Low) |  |  |  |
| Medium (0.55-0.699) |  |  | 0.42 [0.40; 0.45]*** |
| High (0.7-0.799) |  |  | 0.43 [0.39; 0.46]*** |
| Very High (0.8-1) |  |  | 0.62 [0.52; 0.75]*** |
| % Villages <200 Pop (<5%) |  |  |  |
| 5-9.99% |  |  | 1.45 [1.39; 1.50]*** |
| >=10% |  |  | 2.01 [1.91; 2.12]*** |
| *p>0.05; **p>0.01; ***p>0.001 |  |  |  |

Online Resource 5: Birth Order and Residence Stratified Logistic Regression Models, Skilled Delivery

|  | **First Order Birth** | | | **Later Order Birth** | | |
| --- | --- | --- | --- | --- | --- | --- |
|  | *Unadjusted* | *Adjusted: Individual* | ***Adjusted: Individual and District*** | ***Unadjusted*** | ***Adjusted: Individual*** | ***Adjusted: Individual and District*** |
|  | N = 49390 | N = 49390 | N = 49390 | N = 92475 | N = 92475 | N = 92475 |
| Disability (no) |  |  |  |  |  |  |
| Yes | 1.13 [0.83; 1.52] | 1.35 [1.00; 1.83] | 1.42 [1.04; 1.93]* | 0.89 [0.78; 1.01] | 1.02 [0.89; 1.16] | 1.03 [0.89; 1.18] |
|  | **Rural** | | | **Urban** | | |
|  | *Unadjusted* | *Adjusted: Individual* | *Adjusted: Individual and District* | *Unadjusted* | *Adjusted: Individual* | *Adjusted: Individual and District* |
|  | N = 122261 | N = 122261 | N = 122261 | N = 19604 | N = 19604 | N = 19604 |
| Disability (no) |  |  |  |  |  |  |
| Yes | 0.91 [0.81; 1.04] | 1.09 [0.96; 1.24] | 1.11 [0.98; 1.26] | 1.00 [0.54; 1.84] | 1.28 [0.69; 2.38] | 1.09 [0.59; 2.04] |

*p>0.05;**p>0.01;***p>0.001

Online Resource 6: Logistic Regression Models, Postnatal Care within 48 Hours

|  | *Unadjusted Model* | *Adjusted Model: Individual* | *Adjusted Model: Individual and District* |
| --- | --- | --- | --- |
|  | N=141018 | N=141018 | N=141018 |
| *Predictor Variable* |  |  |  |
| Disability (no) |  |  |  |
| Yes | 0.87[0.78;0.96]** | 1.04[0.93;1.16] | 1.03[0.93;1.15] |
| *Individual Covariates* |  |  |  |
| Age at Birth (20-34) |  |  |  |
| 15-19 |  | 0.87[0.82;0.92]*** | 0.86[0.82;0.91]*** |
| 30-34 |  | 1.23[1.16;1.29]*** | 1.23[1.17;1.30]*** |
| Marital Status (Currently Married) |  |  |  |
| Formerly Married |  | 0.96[0.83;1.10] | 0.91[0.79;1.05] |
| Religion (Hindu) |  |  |  |
| Muslim |  | 1.03[0.99;1.08] | 0.98[0.93;1.02] |
| Other |  | 1.06[0.94;1.19] | 0.94[0.83;1.08] |
| Social Group (Other Caste) |  |  |  |
| Scheduled Caste |  | 0.96[0.92;0.99]** | 0.84[0.81;0.87]*** |
| Scheduled Tribe |  | 1.03[1.00;1.07] | 0.99[0.95;1.02] |
| Highest Education (No education) |  |  |  |
| Up to Primary |  | 1.50[1.45;1.55]*** | 1.47[1.42;1.52]*** |
| Middle |  | 2.11[2.01;2.21]*** | 1.98[1.89;2.08]*** |
| Class 10 or 12 |  | 2.86[2.68;3.04]*** | 2.60[2.44;2.78]*** |
| Higher Education |  | 4.59[4.13;5.10]*** | 3.73[3.35;4.16]*** |
| Parity |  | 0.88[0.87;0.89]*** | 0.88[0.87;0.89]*** |
| *District Covariates* |  |  |  |
| Residence (Rural) |  |  |  |
| Urban |  |  | 1.57[1.50;1.65]*** |
| Sex Ratio Categories (High) |  |  |  |
| Low |  |  | 1.00[0.96;1.04] |
| Population Served per Medical Institution (3000-3499) |  |  |  |
| 3500-3999 |  |  | 2.34[2.21;2.47]*** |
| 4000-4499 |  |  | 2.83[2.66;3.01]*** |
| 4500-4999 |  |  | 2.46[2.32;2.61]*** |
| 5000+ |  |  | 3.01[2.80;3.24]*** |
| HDI Score Category (Low) |  |  |  |
| Medium (0.55-0.699) |  |  | 0.39[0.37;0.41]*** |
| High (0.7-0.799) |  |  | 0.40[0.38;0.43]*** |
| Very High (0.8-1) |  |  | 0.29[0.25;0.33]*** |
| % Villages <200 Pop (<5%) |  |  |  |
| 5-9.99% |  |  | 1.48[1.43;1.53]*** |
| >=10% |  |  | 1.73[1.65;1.80]*** |
| *p>0.05; **p>0.01; ***p>0.001 |  |  |  |

Online Resource 7: Birth Order and Residence Stratified Logistic Regression Models, Postnatal Care within 48 Hours

|  | **First Order Birth** | | | **Later Order Birth** | | |
| --- | --- | --- | --- | --- | --- | --- |
|  | *Unadjusted* | *Adjusted: Individual* | *Adjusted: Individual and District* | *Unadjusted* | *Adjusted: Individual* | *Adjusted: Individual and District* |
|  | N=49082 | N=49082 | N=49082 | N=91936 | N=91936 | N=91936 |
| Disability (no) |  |  |  |  |  |  |
| Yes | 1.27[0.99;1.65] | 1.45[1.12;1.88]** | 1.47[1.13;1.91]** | 0.83[0.73;0.93]** | 0.92[0.82;1.04] | 0.92[0.81;1.04] |
|  | **Rural** | | | **Urban** | | |
|  | *Unadjusted* | *Adjusted: Individual* | *Adjusted: Individual and District* | *Unadjusted* | *Adjusted: Individual* | *Adjusted: Individual and District* |
|  | N=121472 | N=121472 | N=121472 | N=19546 | N=19546 | N=19546 |
| Disability (no) |  |  |  |  |  |  |
| Yes | 0.89[0.79;0.99]* | 1.02[0.91;1.14] | 1.02[0.91;1.14] | 1.02[0.65;1.60] | 1.25[0.79;1.97] | 1.16[0.73;1.83] |

*p>0.05; **p>0.01; ***p>0.001
